# Supplementary material for: Quantitative Detection of Natural Rubber Content in Eucommia ulmoides by Portable Pyrolysis-Membrane Inlet Mass Spectrometry
Source: Molecules. 2023 Apr 10;28(8):3330. doi: 10.3390/molecules28083330 (PMC10142753; doi:10.3390/molecules28083330)
Supplement: Supplementary file 1 [file molecules-28-03330-s001.zip › molecules-2282781-supplementary.pdf]

molecules

Article

Supplementary Material

Quantitative Detection of Natural Rubber Content in *Eucommia ulmoides* by Portable Pyrolysis-Membrane Inlet Mass Spectrometry

Minmin Guo [1,2], Mingjian Zhang [1,2], Shunkai Gao [1,2], Lu Wang [1,2], Jichuan Zhang [2,3], Zejian Huang [4,*] and Yiyang Dong [1,2*]

* Corresponding author:

Zejian Huang

huangzj@nim.ac.cn

* Corresponding author:

Yiyang Dong

yydong@mail.buct.edu.cn

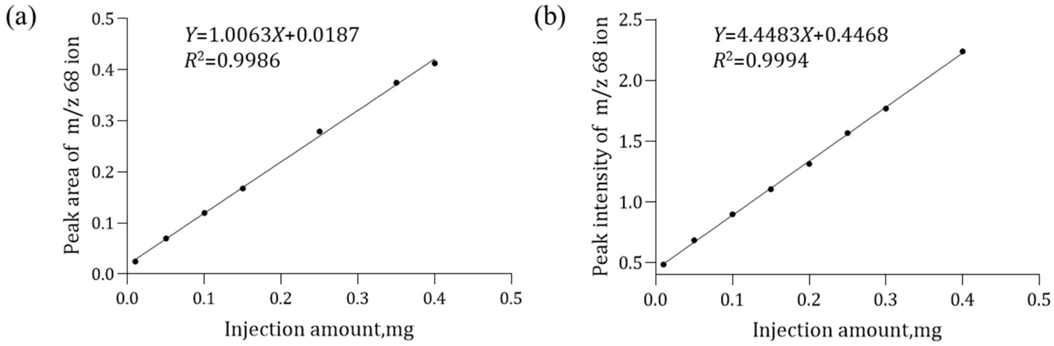

Figure S1. (**a**) Calibration curve based on *m*/*z* 68 ion peak area; (**b**) Calibration curve based on *m*/*z* 68 ion peak intensity.

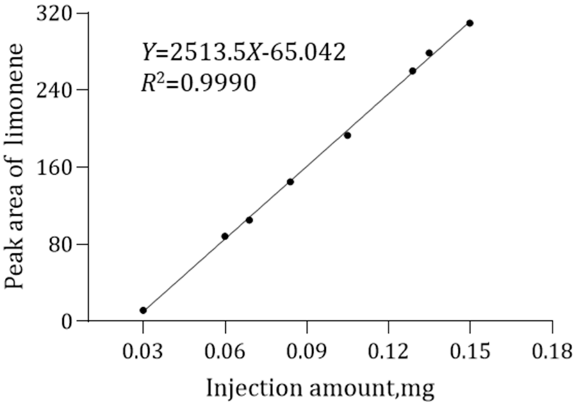

Figure S2. Calibration curve of limonene by PY-GC method.
